# Supplementary material for: Conformational Equilibria in Monomeric α-Synuclein at the Single-Molecule Level
Source: PLoS Biol. 2008 Jan 15;6(1):e6. doi: 10.1371/journal.pbio.0060006 (PMC2174973; doi:10.1371/journal.pbio.0060006)
Supplement: Supplementary file 1 [file pbio.0060006.sd001.doc]

**Conformational Equilibria of Monomeric α**-**Synuclein at the Single Molecule Level**

Massimo Sandal, Francesco Valle, Isabella Tessari, Stefano Mammi, Elisabetta Bergantino, Francesco Musiani, Marco Brucale, Luigi Bubacco, and Bruno Samorì

**SUPPLEMENTARY MATERIAL**

**Figure S1**. (top panel) Schematic diagram of pAFM3s3 vector obtained from cloning αSyn sequence in J. Clarke’s pRSET A modified vector and (bottom panel) representation of the chimeric protein 3S3 coded by the cloned DNA sequence. Titin module numbers refers to the original vector described in the work of Steward et. al [1]. pAFM (I27)3mer and pAFM1s1 vectors and corresponding chimeric protein (respectively 3T, 1S1) can be described with similar diagrams (see article text).

**Figure S2**. Sample of purified 3S3, SDS-10% PAGE and Western blot analysis. **MW**, molecular mass markers (Amersham Biosciences); **lane a**, final product of immobilized metal affinity chromatography (IMAC) purification of 3S3 chimeric protein; **lane b**, Western blotting with anti-His tag Ab; **lane c**, Western blotting with anti-αSyn Ab. The arrow indicate a band corresponding to 3S3 protein with an expected molecular weight of 78119 Da which is in good agreement with observed electrophoretical mobility. The lower band that is copurified with 3S3 is recognised only by the anti-His tag Ab, indicating that it is probably an abortive product of translation that does not contain αSyn and, consequently, the last three titin modules with the two final cysteines, necessary for linking the protein to the gold surface. The lower band recognised in lane c is present also in negative controls (data not shown) so it can be considered an aspecific band.

**Figure S3. Gaussian kernel density estimation of the Mechanically Weak Interaction lengths under different conditions.** Briefly, a Gaussian function (kernel) has been centered on each data point for each data set. The sum of the kernels,normalized to have unitary integral, is the KDE plot. Kernel bandwidth *h* **(**i.e. the Gaussian kernel standard deviation) was automatically calculated for each data set such that it minimizes the asymptotic mean integrated square error: $\text{h=σ}\left( \frac{4}{\text{3n}} \right)^{\left( \frac{1}{5} \right)}$, where σ is the standard deviation of data and *n* is the size of the data set. The plot has been calculated using Statistics for Python (http: //bonsai.ims.u-tokyo.ac.jp/~mdehoon/software/python/Statistics/).

**Figure S4**. **An electrostatic model that may explain mechanically weak interactions.** (**A**) Schematic representation of the central portion of 3S3 construct, evidencing the I27 modules flanking the αSyn element (named I27N and I27C, see text). In the αSyn moiety three regions are evidenced: i) the amphipatic region, prone to fold in α-helical structures when in contact with phospholipid membranes; ii) the fibrillogenic NAC region, characteristic of the fibrils core of αSyn amyloid; and iii) the acidic C-terminal tail, strongly charged and not prone to assume any fold. The reported quotes correspond to interactions that may lead to the small peaks observed in curves featuring mechanically weak interactions (see text). The colors of the different regions depict the electrostatic potential: *red* for the negatively charged I27 (see text) and for the acidic C-terminal region of αSyn; *white* for the hydrophobic NAC region of αSyn; and *blue* for the positively charged amphipatic region of αSyn. (**B**) Cartoon (left panels) and solid surface representations of the electrostatic potential (central and right panels) of titin I27 domain obtained using the program DeepView [2]. I27 coordinates were taken from the Protein Data Bank (PDB code: 1WAA). In the cartoon, the secondary structure elements are colored in *blue* for β-strands. Surfaces were colored according to the calculated electrostatic potential contoured from -5.0 *k*T/*e* (*intense red*) to +5.0 (where *k* = Boltzman constant, T = absolute temperature, and *e* = electron charge) (*intense blue*). The molecular orientation in the central panel is the same as that in the cartoon (left panel), whereas that in the right panel is rotated by 180° about the vertical axis.

**Figure S5**. **Mixing αSyn and I27 in solution does not induce αSyn helical folding.** CD spectra of: the 3T construct in PBS 1X e glycerol 15% (red), αSyn in PBS 1X e glycerol 15% (black), a mixture of 3T and αSyn (green), a mixture of the two protein without the 3T contribution (blue). αSyn concentration was 10 μM and the ratio with the 3T construct was adjusted in order to have a ratio of one αSyn per two I27 modules. The spectra indicate that to induce the helical conformation in αSyn by I27 a close and constrained contact is necessary. It is very difficult to obtain this very high local concentration condition in a mixing experiment.

**Protocol S1. Supplementary Results and Discussion.**

**The distribution of mechanically weak interaction lengths is differently affected by variations in the environment or protein sequence.**

We characterized the mechanically weak interactions (see text, Fig.1D) by measuring the distance between the contour length of events attributed to them and the contour length measured on the first I27 peak in the same curves, both obtaind by a double-parameter WLC fit. This value should correspond to the chain segment length enclosed by the mechanically weak interactions . The larger set of mechanically weak interactions events is that obtained on Tris 10 mM (*nMWI*_Tris 10mM_ = 19). Data recorded in other conditions show a significant decrease in the occurrence of mechanically weak events. The resulting data sets are too small for a significant statistical comparison to be made for each condition, but allow to visualize general trends.

We tried to understand the underlying trends by using kernel density estimation (KDE) [3] to obtain a sketch of the probability density function of interaction lengths. This refined method does not suffer of biases in the choice of binning boundaries and allows for a more objective visualization of small data sets than a classical histogram.

The KDE plot (Supplementary Material Figure 3) of the interaction lengths found in Tris 10 mM and the Tris 10 mM + 1 µM CuCl_2_ for mechanically weak interactions events are almost perfectly superimposable, despite the difference in the size of the data set (*nMWI*_Cu 1 µM_ = 5). The larger number of data in plain Tris 10 mM allows nonetheless to distinguish a bimodal distribution distinguishing mechanically weak interactions enclosing loops of 11 and 25 nm. Experiments conducted with 500 mM buffer concentration (*nMWI*_Tris 500 mM_ = 8) and the A30P mutant (*nMWI*_A30P_ = 6) show instead a broadening of the KDE and allow to infer a shift from short- to long-distance interactions.

These preliminary data seem to indicate an increase of the conformational freedom of the correspondent architectures. This is in line with the observed increased flexibility of the αSyn chain in presence of the A30P mutation [4] . Instead, as previously discussed, in the case of added Cu^2+^ ions, the reason for the increase of β-like structures lies most probably in the stabilization of the metal binding interface centered around His 50, and not on a broad effect on the protein flexibility. However larger data sets will be required to confirm these conclusions.

## A model of the mechanically weak interactions

Considering electrostatic and hydrophobic features of the different construct domains, we can try, very tentatively, to associate the mechanically weak interactions with those of different domains within the 3S3 construct. We can sketch and divide the overall structure of αSyn into three main regions: the amphipatic N-terminal tail (aa 1-61), positively charged because of its Lys residues; a hydrophobic central (aa 61-95) region containing the fibrillogenic NAC segment; and the highly negatively charged acidic C-terminal tail. In the 3S3 construct the αSyn N- and C- termini are flanked by partially negatively charged I27 modules (Fig. 5) [5], that we label I27N and I27C, respectively. Contacts between the negatively charged region of I27N and the positive αSyn N-terminal would appear as peaks that correspond to short-range distances along the primary structure: lower than or equal to 21 nm. The peaks in the middle-range distance of 21-33 nm, and those in the long-range distance of 27-49 nm might correspond to contacts of I27N with the central region of αSyn, and to contacts of the αSyn N-terminal with either the I27C or the αSyn C-terminal, respectively.

The increase of the electrostatic shielding with the buffer concentration has two main effects. First, the population of the mechanically weak interaction featuring structures decreases from about 55% to 23% (see text, Fig. 2), most likely because the interactions with the I27 domains that drive αSyn towards α-helical structures are less favored. Second, the rigidity of the C-terminal tail is decreased as also evidenced by the shift from the short- to the long-distance intermolecular interactions with the increase of the buffer concentration (see Supplementary Material Figure 3). This reduction of rigidity is associated to an increased conformational freedom of the whole αSyn moiety, and most importantly of its NAC region [6].

The intertwining of both effects can shift the conformational equilibrium towards the β-like structures. In fact, their population increases from about 7% to almost 30% on passing from 10 to 500mM Tris/HCl buffer (Fig. 2A).

It is also possible that mechanically weak interaction events contain the signature of the unlocking of αSyn intramolecular interactions. These latter interactions can sustain the fairly compact structures reported by many authors already [6-11], even if their distribution is in our experiments affected by the insertion of the αSyn moiety in the 3S3 construct. For instance the electrostatic repulsion between the negatively charged I27 modules should hinder the long-range contacts between the C- and N-terminal domains proposed by several authors (see below, and Fig. 5) [8, 9].

**Supplementary References:**

[1]	Steward, A., J.L. Toca-Herrera, and J. Clarke, *Versatile cloning system for construction of multimeric proteins for use in atomic force microscopy.* Protein Sci, 2002. **11**(9): p. 2179-83.

[2]	Guex, N. and M.C. Peitsch, *SWISS-MODEL and the Swiss-PdbViewer: an environment for comparative protein modeling.* Electrophoresis, 1997. **18**(15): p. 2714-23.

[3]	Parzen, E., *On Estimation of a Probability Density Function and Mode.* Ann. of Math. Stat., 1962. **33**(3): p. 1065-1076

[4]	Bertoncini, C.W., C.O. Fernandez, C. Griesinger, T.M. Jovin, and M. Zweckstetter, *Familial mutants of alpha-synuclein with increased neurotoxicity have a destabilized conformation.* Journal of Biological Chemistry, 2005. **280**(35): p. 30649-30652.

[5]	Lu, H., B. Isralewitz, A. Krammer, V. Vogel, and K. Schulten, *Unfolding of titin immunoglobulin domains by steered molecular dynamics simulation.* Biophys J, 1998. **75**(2): p. 662-71.

[6]	Fernandez, C.O., W. Hoyer, M. Zweckstetter, E.A. Jares-Erijman, V. Subramaniam, C. Griesinger, and T.M. Jovin, *NMR of alpha-synuclein-polyamine complexes elucidates the mechanism and kinetics of induced aggregation.* Embo J, 2004. **23**(10): p. 2039-46.

[7]	Lee, J.C., R. Langen, P.A. Hummel, H.B. Gray, and J.R. Winkler, *Alpha-synuclein structures from fluorescence energy-transfer kinetics: implications for the role of the protein in Parkinson's disease.* Proc Natl Acad Sci U S A, 2004. **101**(47): p. 16466-71.

[8]	Bernado, P., C.W. Bertoncini, C. Griesinger, M. Zweckstetter, and M. Blackledge, *Defining long-range order and local disorder in native alpha-synuclein using residual dipolar couplings.* J Am Chem Soc, 2005. **127**(51): p. 17968-9.

[9]	Bertoncini, C.W., Y.S. Jung, C.O. Fernandez, W. Hoyer, C. Griesinger, T.M. Jovin, and M. Zweckstetter, *Release of long-range tertiary interactions potentiates aggregation of natively unstructured alpha-synuclein.* Proc Natl Acad Sci U S A, 2005. **102**(5): p. 1430-5.

[10]	Dedmon, M.M., K. Lindorff-Larsen, J. Christodoulou, M. Vendruscolo, and C.M. Dobson, *Mapping long-range interactions in alpha-synuclein using spin-label NMR and ensemble molecular dynamics simulations.* J Am Chem Soc, 2005. **127**(2): p. 476-7.

[11]	Dusa, A., J. Kaylor, S. Edridge, N. Bodner, D.P. Hong, and A.L. Fink, *Characterization of oligomers during alpha-synuclein aggregation using intrinsic tryptophan fluorescence.* Biochemistry, 2006. **45**(8): p. 2752-60.
